# PPAR Agonist-Induced Reduction of Mcp1 in Atherosclerotic Plaques of Obese, Insulin-Resistant Mice Depends on Adiponectin-Induced Irak3 Expression

**DOI:** 10.1371/journal.pone.0062253

**Published:** 2013-04-19

**Authors:** Maarten Hulsmans, Benjamine Geeraert, Thierry Arnould, Christos Tsatsanis, Paul Holvoet

**Affiliations:** 1 Atherosclerosis and Metabolism Unit, Department of Cardiovascular Sciences, KU Leuven, Leuven, Belgium; 2 Laboratory of Biochemistry and Cell Biology (URBC), NAmur Research Institute for LIfe Sciences (NARILIS), University of Namur (FUNDP), Namur, Belgium; 3 Department of Clinical Chemistry, School of Medicine, University of Crete, Heraklion, Greece; University of Padova, Italy

## Abstract

Synthetic peroxisome proliferator-activated receptor (PPAR) agonists are used to treat dyslipidemia and insulin resistance. In this study, we examined molecular mechanisms that explain differential effects of a PPARα agonist (fenofibrate) and a PPARγ agonist (rosiglitazone) on macrophages during obesity-induced atherogenesis. Twelve-week-old mice with combined leptin and LDL-receptor deficiency (DKO) were treated with fenofibrate, rosiglitazone or placebo for 12 weeks. Only rosiglitazone improved adipocyte function, restored insulin sensitivity, and inhibited atherosclerosis by decreasing lipid-loaded macrophages. In addition, it increased *interleukin-1 receptor-associated kinase-3* (*Irak3*) and decreased *monocyte chemoattractant protein-1* (*Mcp1*) expressions, indicative of a switch from M1 to M2 macrophages. The differences between fenofibrate and rosiglitazone were independent of *Pparγ* expression. In bone marrow-derived macrophages (BMDM), we identified the rosiglitazone-associated increase in adiponectin as cause of the increase in Irak3. Interestingly, the deletion of Irak3 in BMDM (IRAK3^−/−^ BMDM) resulted in activation of the canonical NFκB signaling pathway and increased Mcp1 protein secretion. Rosiglitazone could not decrease the elevated Mcp1 secretion in IRAK3^−/−^ BMDM directly and fenofibrate even increased the secretion, possibly due to increased mitochondrial reactive oxygen species production. Furthermore, aortic extracts of high-fat insulin-resistant LDL-receptor deficient mice, with lower *adiponectin* and *Irak3* and higher *Mcp1*, showed accelerated atherosclerosis. In aggregate, our results emphasize an interaction between PPAR agonist-mediated increase in adiponectin and macrophage-associated Irak3 in the protection against atherosclerosis by PPAR agonists.

## Introduction

Low-grade chronic inflammation is associated with obesity and obesity-induced metabolic disorders such as insulin resistance, type 2 diabetes, the metabolic syndrome and atherosclerosis [1], [2]. The recruitment of monocytes and differentiation and polarization to classically activated pro-inflammatory M1 macrophages instead of anti-inflammatory M2 macrophages, have been causally linked to the development of adipose tissue dysfunction, the metabolic syndrome and atherosclerosis [3]. The monocyte chemoattractant protein-1 (Mcp1, also known as chemokine (C-C motif) ligand-2, Ccl2), a marker of M1 macrophages, increases macrophage infiltration, inflammation and insulin resistance in transgenic mice. Conversely, disruption of Mcp1 or its receptor Ccr2 impairs migration of macrophages thereby lowering adipose tissue inflammation and improving insulin sensitivity [4]. It is also recognized that maladaptive production of various adipocytokines (e.g. adiponectin, resistin, visfatin, and leptin) and pro-inflammatory cytokines, such as tumor necrosis factor-α (TNFα) and interleukin (IL)-6, are implicated in the development of obesity-related systemic inflammation and insulin resistance. In particular, the blood levels of adiponectin are significantly lower in obese individuals and have been associated with metabolic inflammation, insulin resistance and the development of cardiovascular disease [5]. The protective effect of adiponectin has mainly been attributed to its anti-inflammatory action [6]. Interestingly, interleukin-1 receptor-associated kinase-3 (IRAK3; also referred to as IRAK-M), a kinase deficient member of the IRAK family and an important negative regulator of toll-like receptor/nuclear factor κB (NFκB) signaling [7], [8], is a major mediator of globular adiponectin-induced endotoxin tolerance in macrophages [9]. Recently, we showed that decreased expression of *IRAK3* in monocytes of obese patients is associated with a high prevalence of metabolic syndrome; weight loss results in an increase in *IRAK3* that is associated with decreased systemic inflammation [10].

Several clinical trials support the use of peroxisome proliferator-activated receptor (PPAR) agonists to treat dyslipidemia and insulin resistance in obesity and type 2 diabetes. PPARα agonists such as fenofibrate regulate lipoprotein metabolism [11]. PPARγ agonists such as rosiglitazone reduce blood glucose levels in patients with type 2 diabetes [12]. Recent data also suggest critical roles of PPAR agonists in inhibiting vascular inflammation and atherosclerosis [13]–[15]. However, it remains to be determined if molecular mechanisms that confer their vascular protection are identical for PPARα and PPARγ agonists.

In the present study, we aimed to analyze the consequences of PPARα and PPARγ agonist treatment on macrophage activation in relation to atherogenesis using mice with combined leptin (Ob/Ob) and LDL-receptor deficiency (double knockout [DKO] mice). These mice are characterized by morbid obesity, dyslipidemia, glucose intolerance and insulin resistance, and accelerated atherosclerosis [16], [17]. Atherosclerotic lesions cover 20% of total area of the thoracic abdominal aorta in double-mutant mice compared with 3.5% in LDL-receptor deficient mice. Ob/Ob mice have no detectable lesions. Higher macrophage homing is detected prior to increase in plaque volumes in the aortic root of DKO mice [16]. In intra-abdominal adipose tissues of DKO mice, Pparγ expression is lower than in LDL-receptor deficient and Ob/Ob mice, and Pparα expression is lower than in LDL-receptor deficient mice. Weight loss increases Pparα and Pparγ, associated with a decrease in atherosclerosis [17]. Because DKO mice, in contrast to single KO mice, are characterized by decreases in Pparα and Pparγ and accelerated atherosclerosis, we selected DKO mice to investigate the effect of fenofibrate and rosiglitazone. We found that rosiglitazone treatment in contrast to fenofibrate increases systemic adiponectin levels and *Irak3* expression, resulting in decreased expression of *Mcp1* in atherosclerotic plaques. Moreover, the presence of Irak3 in macrophages is necessary for the indirect Mcp1-reducing effects of PPAR agonists. Because defective leptin signaling was found to modulate inflammation and atherosclerosis [18], we also studied the relation between low *adiponectin* (*Adipoq*) and *Irak3* expression in aortic extracts of high-fat and insulin resistant LDL-receptor deficient mice characterized by high plasma leptin levels.

## Materials and Methods

### Experimental Protocol of Animal Studies

**Figure 1:**
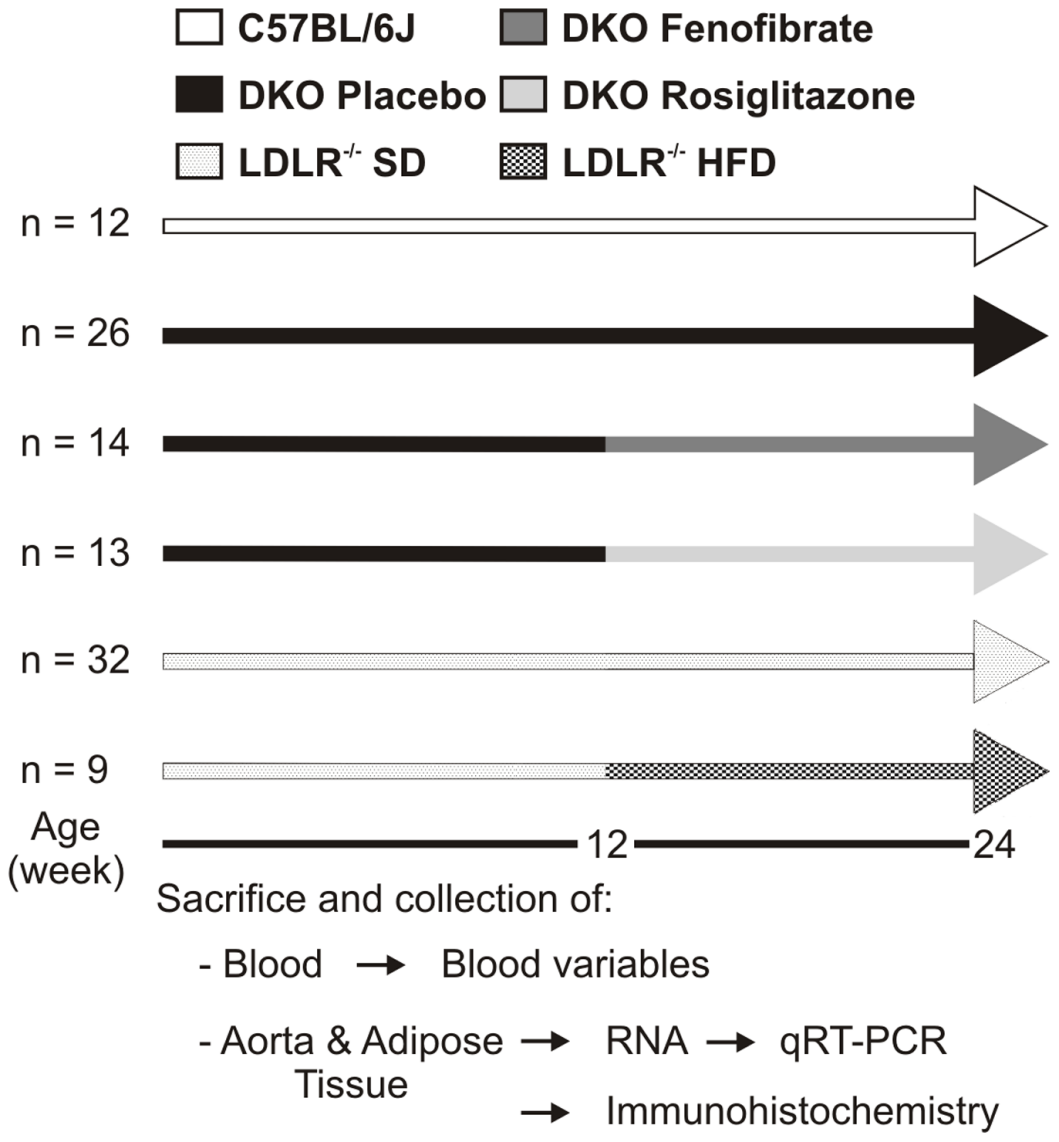
Experimental protocol. Twelve-week old DKO mice were treated for 12 weeks with fenofibrate (50 mg kg^−1^ day^−1^) or with rosiglitazone (10 mg kg^−1^ day^−1^) and were compared with placebo-treated DKO and C57BL/6 J background mice. LDL-receptor deficient (LDLR^−/−^) mice, on the C57BL/6 J background, were fed for a period of 12 weeks with standard diet (SD) or with a high-fat diet (HFD). The mice were sacrificed at 24 weeks and total blood, the aortic arch, the abdominal aorta, and visceral adipose tissue were collected.

Animal experiments conformed to the Guide for the Care and Use of Laboratory Animals published by the US National Institutes of Health. They were approved by the Institutional Animal Care and Research Advisory Committee of the KU Leuven (Permit Number: P087/2007). Breeding and genotyping of DKO mice, on the C57BL/6 J background, were performed as previously described [16], [17]. Figure 1 shows a schematic diagram of the experimental protocol. For comparison, age- and gender-matched lean C57BL/6 J mice (n = 12) were used. Fenofibrate and rosiglitazone (Avandia) were purchased from Sigma-Aldrich and GlaxoSmithKline. DKO mice were treated with fenofibrate (n = 14), rosiglitazone (n = 13) or placebo (n = 26) for 12 weeks starting at the age of 12 weeks. Fenofibrate (50 mg kg^−1^ day^−1^) and rosiglitazone (10 mg kg^−1^ day^−1^) were added to standard diet (SD) containing 4% fat (Ssniff), placebo-treated mice received the grinded chow only. Food and water were available *ad libitum*. Food intake of the DKO mice was ≈5.7 g/day and was not affected by the treatments. LDL-receptor deficient mice, on the C57BL/6 J background, were fed *ad libitum* for a period of 12 weeks starting at the age of 12 weeks with SD (n = 32) or with a high-fat diet (HFD) containing 45% fat (n = 9). Food intake was 50% of that of DKO mice, and was not different between SD- and HFD-fed mice. All mice were sacrificed by Nembutal overdose at the age of 24 weeks.

### Blood Values

After an overnight fast, blood was collected by puncturing the *vena cava*. Plasma was obtained by centrifugation. Total cholesterol and triglycerides were measured using standard enzymatic colorimetric assays (Boehringer Mannheim). Glucose was measured with a glucometer (Menarini Diagnostics) and plasma insulin with a mouse insulin enzyme-linked immunosorbent assay (ELISA) (Mercodia). To convert from mg/dL to mM, we divided glucose by 18 ( = molecular weight), triglycerides by 89 and total cholesterol by 39. Insulin resistance was calculated by a homeostasis model assessment of insulin resistance (HOMA-IR) = fasting plasma insulin (mU/L) x fasting blood glucose (mM)/22.5. To determine glucose tolerance, glucose was measured in samples obtained by tail bleeding before and 15, 30, 60, 120 and 240 minutes after intraperitoneal administration of glucose (20% glucose solution; 2 g/kg). Plasma adiponectin, TNFα and IL6 were measured with specific mouse ELISAs (R&D Systems) [19], [20].

### Histological Analysis of Visceral Adipose Tissue and Atherosclerosis

Ten µm-thick paraffin sections of adipose tissue were stained for macrophages with an antibody against mouse Mac-3 antigen (Pharmingen). Blinded analysis was performed on 12 fields of 3 sections from different levels (every 0.1 mm) of the visceral adipose depot for each individual mouse with the Quantimet600 image analyzer (Leica) and a light microscope with 20× magnification. Adipocyte size was determined as the mean adipocyte area calculated by dividing the total adipocyte area by the total number of adipocytes for each mouse. The number of nuclei of Mac-3-positive cells was averaged over 12 different fields, which were most enriched with macrophages, and expressed as ratio to the number of adipocytes. Dead/dying adipocytes were identified by the presence of crown-like structures (CLS). CLS density was obtained by counting the number of CLS in each section and expressed as ratio to the number of adipocytes. Preparation of sections of the aortic valves, determination of the extent of atherosclerosis, and lipid (oil red O) and macrophage (Mac-3 antibody) stainings were performed according to previously described protocols [19], [20].

### Cell Culture

Bone marrow cells were isolated from 24-weeks-old C57BL/6 J, DKO and IRAK3^−/−^ mice. IRAK3^−/−^ mice were kindly provided by Dr. Flavell (Yale University) [8]. After euthanasia, the mice were sprayed with 70% ethanol and the femurs were dissected. Muscles connected to the bone were removed using clean gauze, and the femurs were placed into a Petri dish containing sterile PBS on ice. Both epiphyses were removed using sterile scissors and the bones were flushed with a syringe with DMEM containing 10% FBS (Gibco) to extrude bone marrow. Single cell suspensions were prepared by passing the cells through a 70-µm cell strainer. Cells were washed and incubated in erythrocyte-lysis buffer (Miltenyi) for 10 minutes, according to the manufacturer’s instructions. These fresh bone marrow cells were differentiated to bone marrow-derived macrophages (BMDM) [21], using L929 cell conditioned medium (LCCM) as a source of granulocyte/macrophage colony stimulating factor [22]. The cells derived from one femur were resuspended in 25 mL bone marrow differentiation media, which is DMEM supplemented with 20% FBS, 30% LCCM, 100 U/mL penicillin, 100 mg/mL streptomycin, and 2 mM L-glutamine (Gibco). Cells were seeded in non-tissue culture-treated 150 mm Petri dishes and incubated at 37°C in a 5% CO_2_ atmosphere. Six days after seeding the cells, the attached cells were washed and incubated overnight under normal growth conditions. Then, cells were incubated with 50 µM fenofibrate, 10 µM rosiglitazone, 5 µM GW9662 (Sigma-Aldrich) and 1 or 10 µg/mL murine globular adiponectin (PeproTech) for 24 hours. Cell viability, as determined by trypan blue exclusion, was >80%.

### RNA Extraction and Gene Expression Analysis

Total *RNA* was extracted with TRIzol reagent (Invitrogen) and purified on RNeasy Mini kit columns (Qiagen). First-strand cDNA was generated from total *RNA* with the SuperScript VILO cDNA synthesis kit (Invitrogen). Quantitative real-time PCR (qRT-PCR) was performed using Fast SYBRGreen master mix according to the supplier’s protocol (Applied Biosystems). Oligonucleotides (Invitrogen) used as forward and reverse primers were designed using the Primer Express software (Applied Biosystems) and are summarized in Table 1. Data were normalized to the housekeeping gene *β-actin* as previously described [20].

**Table 1:** Primers used for qRT-PCR analysis.

Gene symbol	Forward primer	Reverse primer
*Adipoq*	5′-CTCCTCATTTCTGTCTGTACGATTG-3′	5′-ACAGTAGCATCCTGAGCCCTTT-3′
*β-actin*	5′-ACGGCCAGGTCATCACTATTG-3′	5′-CACAGGATTCCATACCCAAGAAG-3′
*IL6*	5′-CTGTTGGGAGTGGTATCCTCTGT-3′	5′-CCACGGCCTTCCCTACTTC-3′
*Irak3 or Irak-m*	5′-TTTGCTAGGTTTTGGTTGTCAGAA-3′	5′-TGAGAAGCTAAACTGGAGCCAAT-3′
*Mcp1 or Ccl2*	5′-GCAGTTAACGCCCCACTCA-3′	5′-CAGCCTACTCATTGGGATCATCTT-3′
*Pparα*	5′-TCAGGGTACCACTACGGAGTTCA-3′	5′-CCGAATAGTTCGCCGAAAGA-3′
*Pparγ*	5′-GCAGCTACTGCATGTGATCAAGA-3′	5′-GTCAGCGGGTGGGACTTTC-3′
*Tnfα*	5′-GGGAGGCCATTTGGGAACT-3′	5′-GCCACCACGCTCTTCTGTCTA-3′

### Western Blotting

Western blot analysis was performed with 20 µg of total protein. Protein was electrophoresed through a 10–20% SDS-polyacrylamide gel (Bio-Rad) and transferred to a polyvinylidene difluoride membrane (Millipore). Membranes were processed according to standard Western blotting procedures. To detect protein levels, membranes were incubated with primary antibodies against β-actin (Cell Signaling Technology) and Irak3 (Rockland Immunochemicals). The membranes were then incubated with horseradish peroxidase-coupled secondary antibody (Santa Cruz Biotechnology) and developed with SuperSignal chemiluminescent substrate (Pierce). A PC-based image analysis program was used to quantify the intensity of each band (Bio-1D).

### Soluble Mcp1 Cytokine Levels

Conditioned medium was harvested after treatment of BMDM and stored at −80°C. Soluble Mcp1 protein levels in the conditioned medium were determined by ELISA according to the manufacturer’s instructions (R&D Systems).

### NFκB p50 DNA Binding Activity

NFκB p50 DNA binding activity was assessed on isolated nuclear extracts of BMDM by ELISA using the TransAM NFκB p50 transcription factor assay kit according to the manufacturer’s protocol (Active Motif). Briefly, 10 µg of nuclear extract diluted in complete lysis buffer was used in the p50 binding assay. The samples were shaken for 1 hour at room temperature in 30 µL binding buffer. After washing, anti-p50 antibody diluted 1∶1000 was applied to the wells for 1 hour at room temperature. Specific binding was estimated by spectrophotometry after incubation with a horseradish peroxidaseconjugated antibody (1 hour at room temperature, 1∶1000 diluted) at 450 nm wave length.

### Mitochondrial Reactive Oxygen Species Detection

To detect mitochondrial reactive oxygen species (mROS) formation in treated BMDM, measurements of MitoSOX Red (Invitrogen) fluorescence were performed by flow cytometry (Becton, Dickinson and Company). Cells were incubated with PBS containing 5 µM MitoSOX for 10 minutes at 37°C and 5% CO_2_. The labeled cells were washed twice with PBS and then suspended in warm PBS for analysis by flow cytometry.

### Statistical Analysis

The Kruskal-Wallis nonparametric one-way ANOVA, followed by the Dunn’s multiple comparisons test was used to compare more than two independent samples. The unpaired t-test with Welch’s correction test was used for the two-sample comparisons (Graph Pad Prism version 5). Correlations were calculated using the nonparametric Spearman’s correlation coefficient (r_s_). Nonparametric tests were used because Gaussian distribution cannot be expected in small sample size studies as performed in this article. The area under the curve of the intraperitoneal glucose tolerance test (AUC of IPGTT) was calculated in Graph Pad Prism version 5. A probability value of *P<*0.05 was considered statistically significant.

## Results

### Rosiglitazone in Contrast to Fenofibrate Decreases Systemic Inflammation and Restores Insulin Sensitivity

Body weight, plasma total cholesterol and triglycerides were significantly higher in placebo-treated DKO mice compared with lean C57BL/6 J background mice. Glucose and insulin levels, and thus the HOMA-IR index were similarly elevated. Glucose tolerance as measured by AUC of IPGTT was increased. The higher levels of TNFα and IL6, and the lower adiponectin levels indicated systemic inflammation (Table 2A). Both fenofibrate and rosiglitazone decreased weight and insulin levels. Interestingly, only rosiglitazone treatment reduced glucose levels resulting in a decrease in HOMA-IR and a partial normalization of glucose tolerance. Systemic inflammation was only decreased after rosiglitazone treatment evidenced by lower levels of TNFα and IL6 and increased adiponectin levels. Both treatments had almost no effect on total cholesterol and triglycerides (Table 2A).

**Table 2:** Blood, adipose tissue and plaque variables.

	C57BL/6 J	DKO			ANOVA
		Placebo	Fenofibrate	Rosiglitazone	
**A. Weight and blood variables**					
Weight (g)	28.1±1.0	61.8±0.8***	58.7±1.1*** ^/$^	54.9±1.0*** ^/$$$/£^	*P*<0.001
Total cholesterol (mM)	1.1±0.1	13.4±0.8***	13.7±1.0***	16.6±1.2*** ^/$^	*P*<0.001
Triglycerides (mM)	0.30±0.24	3.1±0.3***	2.4±0.3***	1.9±0.3*** ^/$^	*P*<0.001
Glucose (mM)	4.2±0.2	8.2±0.5***	7.0±0.5***	4.9±0.3* ^/$$$/££^	*P*<0.001
Insulin (mU/L)	74.3±4.4	213.3±24.7***	106.3±5.9*** ^/$$$^	67.1±16.3$$$	*P*<0.001
HOMA-IR	14.2±1.0	90.1±15.1***	31.2±2.2*** ^/$$^	15.8±4.5^$$$/£^	*P*<0.001
AUC of IPGTT	35.1±0.4	87.6±4.9***	74.8±4.2***	50.8±1.2*** ^/$$$/£££^	*P*<0.001
TNFα (pg/mL)	22.4±1.2	29.7±1.9**	29.6±1.0***	24.7±1.3^$/££^	*P*<0.01
IL6 (pg/mL)	17.9±0.6	29.3±2.4***	21.1±2.3$	14.6±1.7^$$$/£^	*P*<0.001
Adiponectin (mg/mL)	5.4±0.6	2.9±0.4**	1.1±0.2*** ^/$$$^	16.2±0.7*** ^/$$$/£££^	*P*<0.001
**B. Adipose tissue variables**					
Adipocyte size (x 10^3^ µm^3^)	2.1±0.2	7.9±0.2***	8.1±0.2***	6.4±0.4*** ^/$$/££^	*P*<0.001
Macrophages/adipocyte	2.8±0.5	219.7±10.8***	122.5±15.3*** ^/$$$^	49.9±3.0*** ^/$$$/£££^	*P*<0.001
CLS (%)	0.08±0.03	10.9±1.1***	5.9±1.3*** ^/$$^	1.3±0.2*** ^/$$$/££^	*P*<0.001
**C. Plaque variables**					
Plaque volume (x 10^−3^ µm^3^)	ND	93.9±5.4	114.6±10.1	60.3±14.2^$/££^	*P*<0.01
Plaque macrophages (% of plaque area)	ND	28.5±2.2	22.5±1.3$	11.6±1.2^$$$/£££^	*P*<0.001
Plaque lipids (% of plaque area)	ND	30.3±1.4	28.4±1.2	24.3±1.2^$$/£^	*P*<0.05

Data are means ± SEM.

* *P<*0.05,

** *P<*0.01 and

*** *P<*0.001 DKO compared with C57BL/6 J mice;

$ *P*<0.05,

$$ *P*<0.01 and

$$$ *P*<0.001 PPAR agonist-treated compared with placebo-treated DKO mice;

£ *P*<0.05,

££ *P*<0.01 and

£££ *P*<0.001 rosiglitazone-treated compared with fenofibrate-treated DKO mice.

Abbreviations: CLS, crown-like structures; ND, not detectable.

### Rosiglitazone and not Fenofibrate Improves Adipocyte Function by Decreasing Macrophage Accumulation

**Figure 2:**
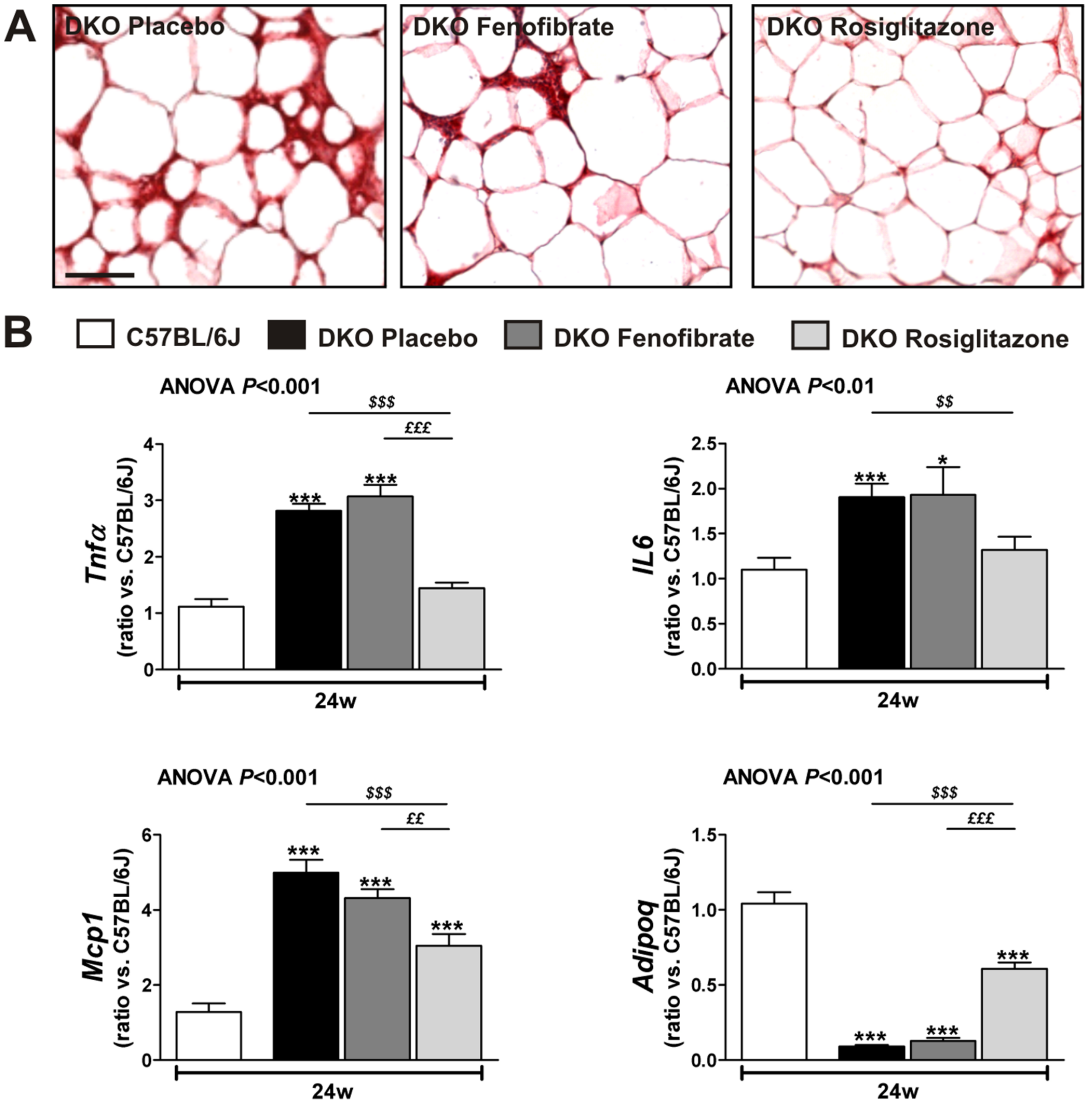
Rosiglitazone and not fenofibrate treatment reduces macrophage accumulation and improves adiponectin expression in visceral adipose tissue. (**A**) Representative Mac-3 staining of visceral adipose tissue of placebo-, fenofibrate- and rosiglitazone-treated DKO mice at 24 weeks. (**B**) Relative *RNA* levels of *Tnfα*, *IL6*, *Mcp1* and *Adipoq* as determined by qRT-PCR. Data are means ± SEM. Scale bar = 100 µm. *P<0.05 and ***P<0.001 DKO compared with C57BL/6 J mice; ^$$^ *P*<0.01 and ^$$$^ *P*<0.001 PPAR agonist-treated compared with placebo-treated DKO mice; ^££^ *P*<0.01 and ^£££^ *P*<0.001 rosiglitazone-treated compared with fenofibrate-treated DKO mice.

Figure 2A shows representative sections of visceral adipose tissue stained for macrophages of placebo-, fenofibrate-, and rosiglitazone-treated DKO mice. The adipocyte size, number of macrophages and CLS, which indicates necrotic adipocytes surrounded by phagocytes, were increased in DKO mice compared with lean C57BL/6 J mice (Table 2B). Only rosiglitazone decreased the adipocyte size. Both treatments, but rosiglitazone more than fenofibrate, reduced the number of macrophages and CLS in adipose tissue of DKO mice (Table 2B and Figure 2A). Furthermore, rosiglitazone decreased *Tnfα*, *IL6* and *Mcp1* expressions, and increased the expression of *Adipoq* (Figure 2B). Rosiglitazone treatment also resulted in a greater increase in *Pparα* than fenofibrate treatment; differences in *Pparγ* were less pronounced (Table 3A).

**Table 3:** *Pparα* and *Pparγ* expressions in visceral adipose and aortic tissues of PPAR agonist-treated DKO mice.

	DKO			ANOVA
	Placebo	Fenofibrate	Rosiglitazone	
**A. Visceral adipose tissue**				
*Pparα*	0.23±0.02***	0.37±0.04*** ^/$$^	1.83±0.17*** ^/$$$/£££^	*P*<0.001
*Pparγ*	0.21±0.01***	0.33±0.03*** ^/$$^	0.43±0.02*** ^/$$$/£^	*P*<0.001
**B. Abdominal aorta**				
*Pparα*	0.58±0.05*	0.57±0.05*	3.33±0.39*** ^/$$$/£££^	*P*<0.001
*Pparγ*	0.67±0.07*	1.00±0.05$$$	1.11±0.09$$$	*P*<0.001

Data are means ± SEM.

* *P<*0.05 and

*** *P<*0.001 DKO compared with C57BL/6 J mice;

$$ *P*<0.01 and

$$$ *P*<0.001 PPAR agonist-treated compared with placebo-treated DKO mice;

£ *P*<0.05 and

£££ *P*<0.001 rosiglitazone-treated compared with fenofibrate-treated DKO mice.

### Rosiglitazone and not Fenofibrate Reduces Atherosclerotic Plaque Volume and Macrophage Content by Decreasing Macrophage Accumulation

**Figure 3:**
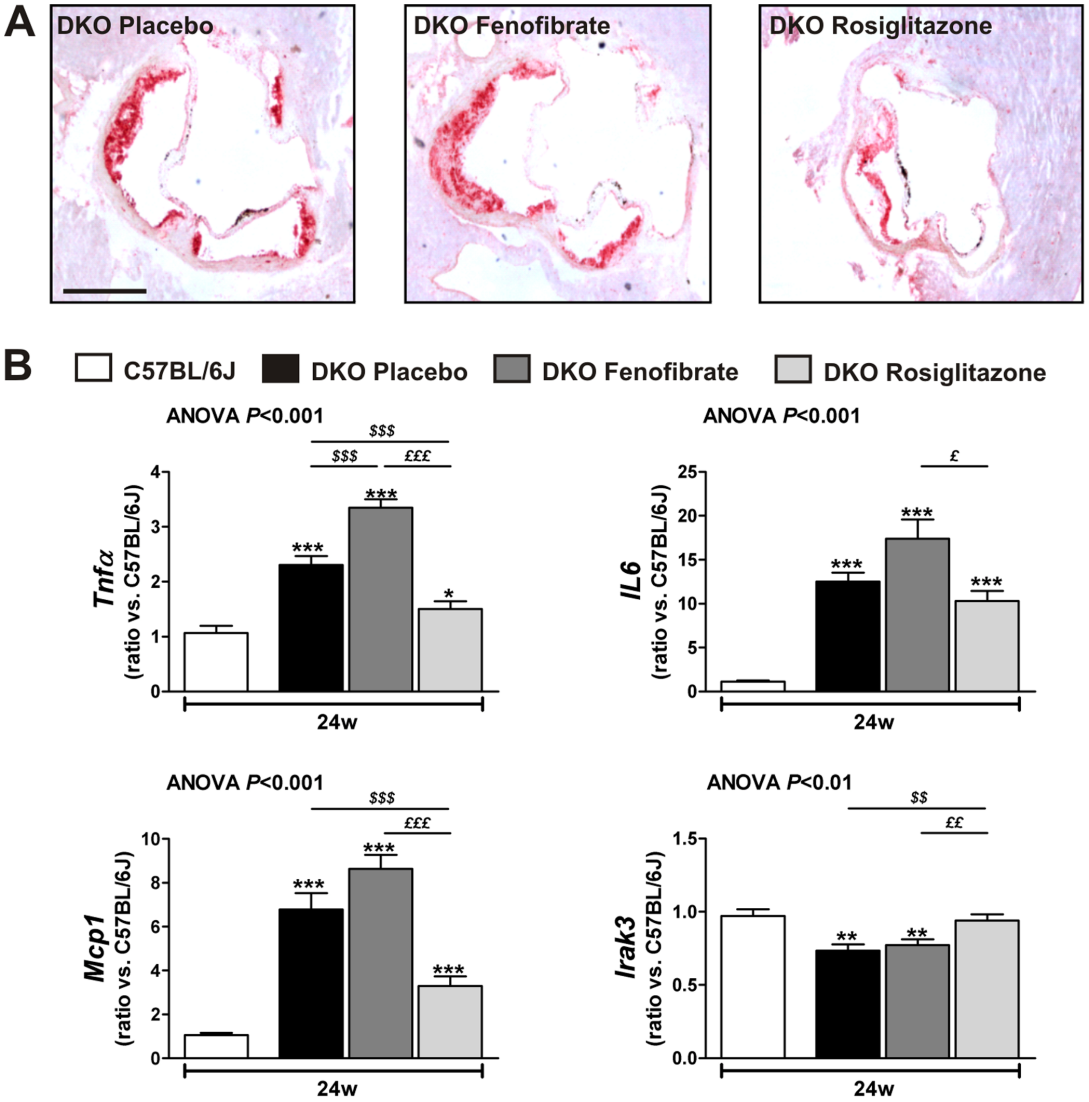
Rosiglitazone and not fenofibrate treatment decreases atherogenesis in obese, insulin resistant mice. (**A**) Representative Mac-3 staining of aortic sinus plaques of placebo-, fenofibrate- and rosiglitazone-treated DKO mice at 24 weeks. (**B**) Gene expression in the aorta was analyzed by measuring relative *RNA* levels using qRT-PCR for *Tnfα*, *IL6*, *Mcp1* and *Irak3*. Data are means ± SEM. Scale bar = 500 µm. *P<0.05, **P<0.01 and ***P<0.001 DKO compared with C57BL/6 J mice; ^$$^ *P*<0.01 and ^$$$^ *P*<0.001 PPAR agonist-treated compared with placebo-treated DKO mice; ^£^ *P*<0.05, ^££^ *P*<0.01 and ^£££^ *P*<0.001 rosiglitazone-treated compared with fenofibrate-treated DKO mice.

Figure 3A shows representative sections of atherosclerotic lesions stained for macrophages of placebo-, fenofibrate-, and rosiglitazone-treated DKO mice. Fenofibrate had no effect on overall plaque volume. In contrast, rosiglitazone decreased plaque volume by inhibiting macrophage and lipid deposition (Table 2C). In addition, rosiglitazone treatment decreased *Tnfα*, *IL6* and *Mcp1* expressions in extracts of abdominal aorta. Furthermore, *Irak3* expression was increased after rosiglitazone treatment (Figure 3B). *Irak3* in the aorta correlated positively with circulating adiponectin levels (r_s_ = 0.41, *P*<0.01) and negatively with *Tnfα* (r_s_ = −0.43, *P*<0.01), *IL6* (r_s_ = −0.35, *P*<0.05) and *Mcp1* (r_s_ = −0.46, *P*<0.001). The expression of *Adipoq* in aortic extracts, 2.1-fold decreased in DKO compared with C57BL/6 J mice (*P*<0.01), increased more after rosiglitazone treatment (1.8-fold *vs.* DKO, *P*<0.01; 1.4-fold *vs.* fenofibrate treatment, *P*<0.05) than fenofibrate treatment (1.2-fold *vs.* DKO, *P*<0.05). Rosiglitazone treatment also resulted in a greater increase in *Pparα* than fenofibrate treatment; differences in *Pparγ* were not significant (Table 3B).

### Irak3 Induction Dependent on High Adiponectin is Required for the Decreased Expression of Mcp1 after Rosiglitazone Treatment

**Figure 4:**
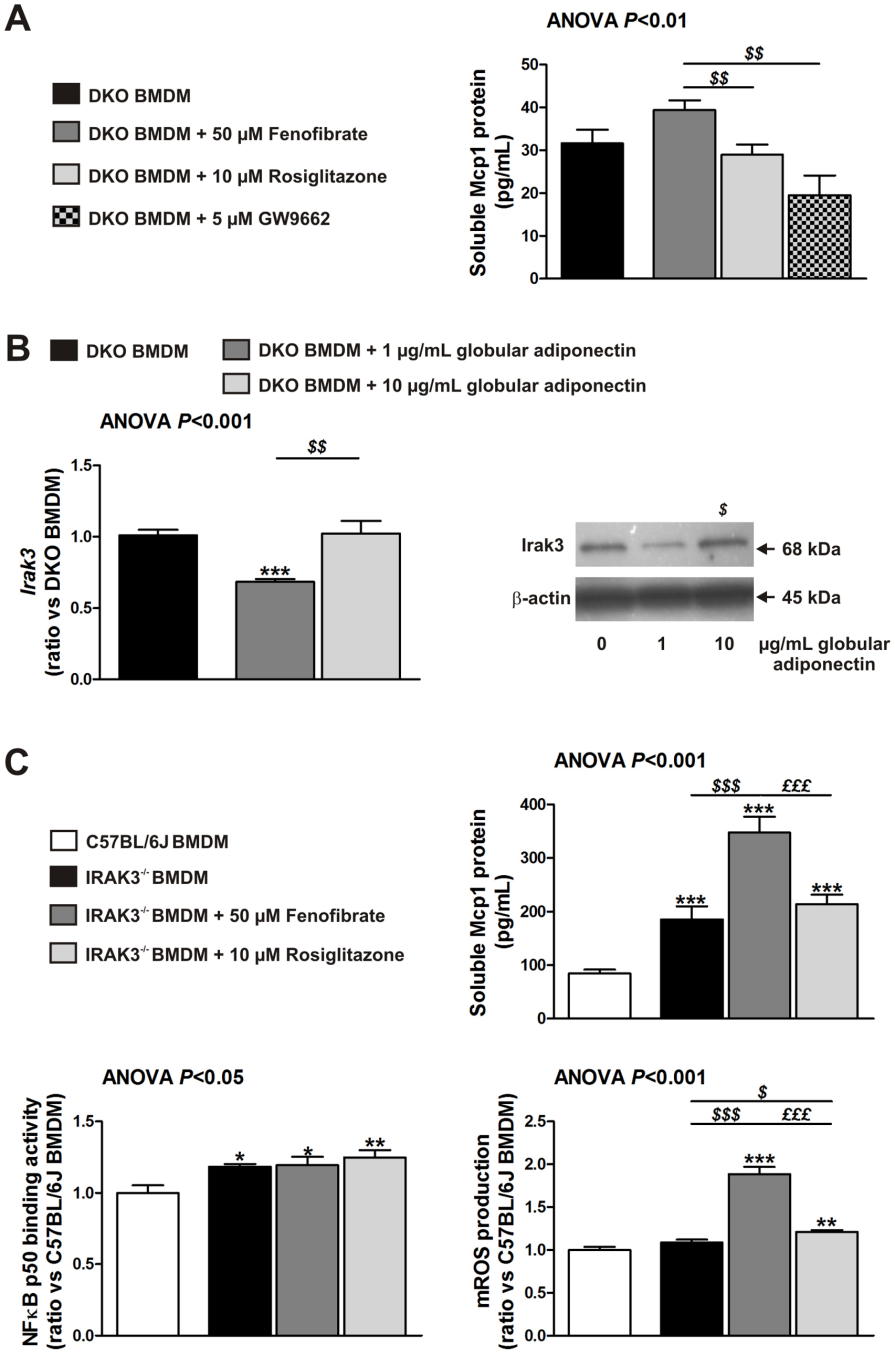
Adiponectin-induced Irak3 plays an important role in rosiglitazone-mediated decrease of Mcp1. (**A**) Soluble Mcp1 protein levels in DKO BMDM exposed to 50 µM fenofibrate, 10 µM rosiglitazone or 5 µM GW9662 for 24 hours as determined by ELISA. Data are means ± SEM; n = 16 from three different mice. ^$$^ *P*<0.01 compared with fenofibrate-treated BMDM. (**B**) Irak3 *RNA* and protein levels of DKO BMDM exposed to 1 or 10 µg/mL globular adiponectin for 24 hours as determined by qRT-PCR and Western blotting. Data are means ± SEM; n = 6. ***P<0.001 compared with DKO BMDM; ^$^ *P*<0.05 and ^$$^ *P*<0.01 compared with DKO BMDM exposed to 1 µg/mL globular adiponectin. (**C**) Soluble Mcp1 protein levels (n = 18 from three different mice), NFκB p50 DNA binding activity (n = 8 from two different mice) and mROS production (n = 6) in IRAK3^−/−^ BMDM exposed to 50 µM fenofibrate or 10 µM rosiglitazone for 24 hours as determined by ELISA and flow cytometry. Data are means ± SEM. *P<0.05, **P<0.01 and ***P<0.001 compared with C57BL/6 J BMDM; ^$^ *P*<0.05 and ^$$$^ *P*<0.001 compared with IRAK3^−/−^ BMDM; ^£££^ *P*<0.001 compared with fenofibrate-treated BMDM. Abbreviations: BMDM, bone marrow-derived macrophages; mROS, mitochondrial reactive oxygen species.

We investigated the direct effect of fenofibrate, rosiglitazone, and PPARγ antagonist (GW9662) treatment on BMDM from DKO mice to elucidate the observed differences between PPAR treatments. Fenofibrate tended to increase Mcp1 protein. Rosiglitazone did not decrease Mcp1 protein and addition of the PPARγ antagonist did not increase Mcp1 protein. In aggregate, we did not observe a direct inhibitory effect of PPAR agonists on Mcp1 protein secretion (average Mcp1 concentration in DKO BMDM: 31.8±1.6 pg/mL) (Figure 4A).

However, we have previously shown that there is a causal relation between variable globular adiponectin levels and IRAK3 expression in blood monocytes of obese patients [10]. Because we found that rosiglitazone increased adiponectin more than fenofibrate, we determined the effect of low (as in untreated and fenofibrate-treated DKO mice) and high (as in rosiglitazone-treated DKO mice) globular adiponectin concentrations on the Irak3 expression in BMDM. Indeed, exposure to 10 µg/mL globular adiponectin increased the Irak3 expression (*RNA* and protein) in comparison with exposure to 1 µg/mL globular adiponectin (Figure 4B).

Then, we investigated if Irak3 deletion had an effect on Mcp1 expression. The deletion of Irak3 in BMDM (IRAK3^−/−^ BMDM) was characterized by activation of the canonical NFκB signaling pathway as determined by NFκB p50 DNA binding activity. This resulted in a 2.2-fold increase in Mcp1 protein secretion compared with control cells (185.5±24.5 *vs.* 84.5±7.3 pg/mL, *P*<0.001). Rosiglitazone treatment did not decrease the elevated Mcp1 secretion in IRAK3^−/−^ BMDM and fenofibrate even increased the production of Mcp1, independently from NFκB p50 DNA binding activity. However, the observed increase in Mcp1 after fenofibrate treatment was associated with increased mROS production (Figure 4C).

### Lower Adipoq and Irak3 Expressions are Associated with M1 Macrophages and Accelerated Atherosclerosis in High-fat Insulin Resistant Mice

**Figure 5:**
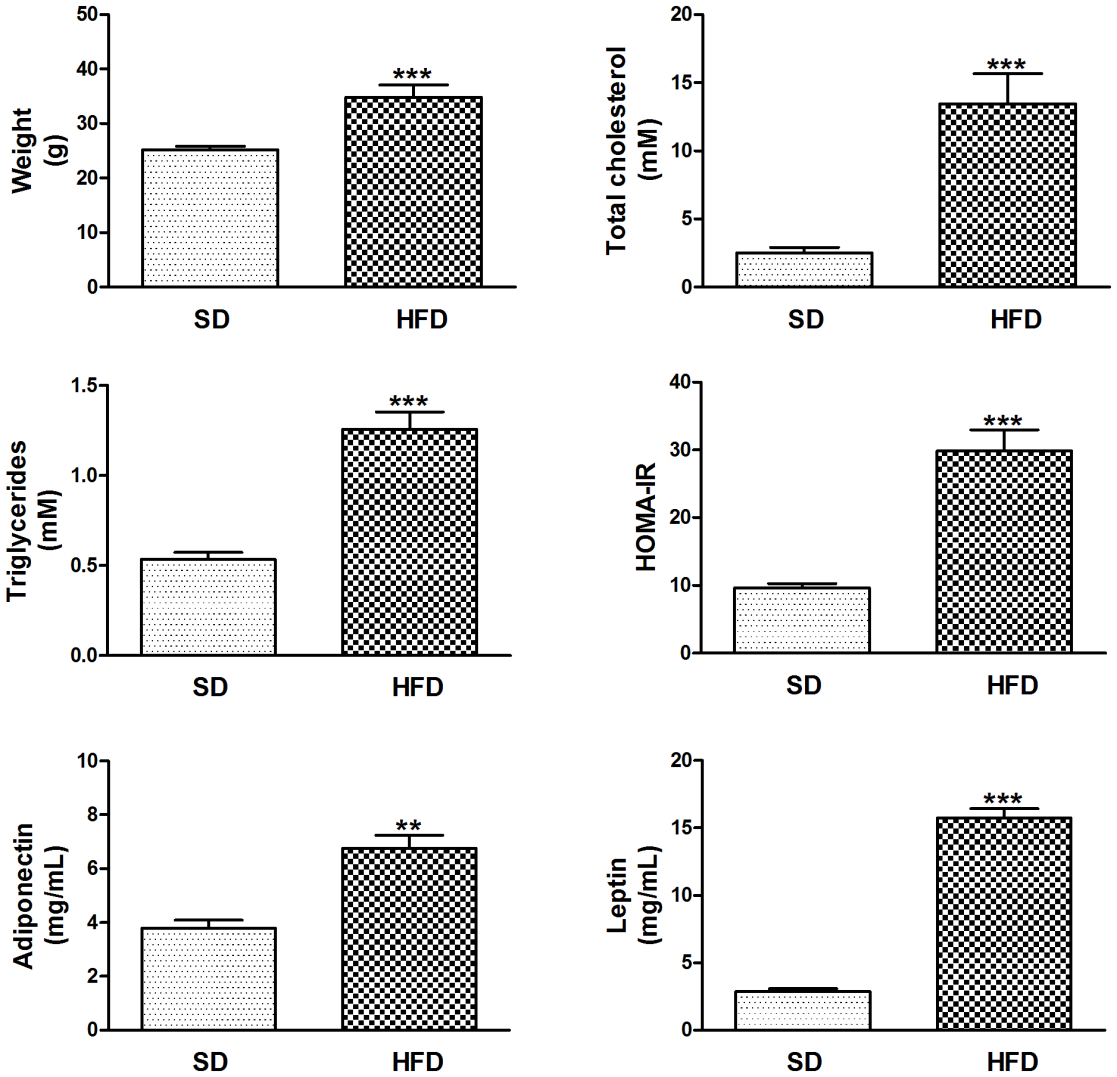
HFD-induced weight gain is associated with dyslipidemia, insulin resistance and hyperleptinemia in the presence of high blood adiponectin. Data are means ± SEM. **P<0.01 and ***P<0.001 HFD-fed compared with SD-fed LDL-receptor deficient mice. Abbreviations: HFD, high fat diet; SD, standard diet.

Body weight, plasma total cholesterol and triglycerides were significantly higher in HFD-fed LDL-receptor deficient mice compared with SD-fed mice, with C57BL/6 J background. Glucose tolerance, as measured by AUC of IPGTT and HOMA-IR index was elevated. In contrast to DKO, HFD-fed LDL-receptor deficient mice had increased blood levels of adiponectin. In addition, their leptin levels were elevated (Figure 5).

**Figure 6:**
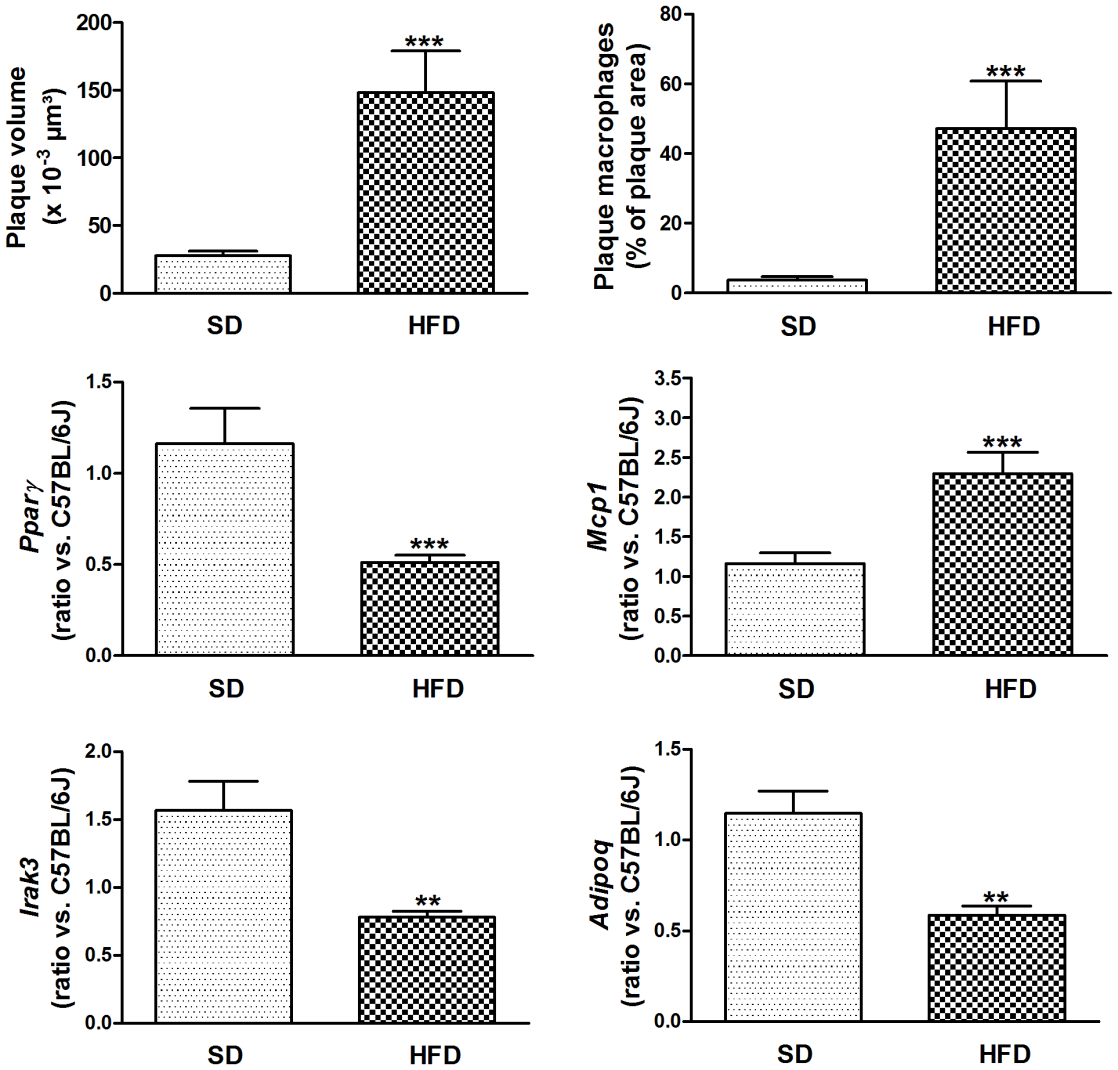
HFD increases atherogenesis in insulin resistant mice. Plaque volume was determined by measuring lipid (oil red O)-stained surfaces in subsequent sections; macrophages were stained with anti-Mac-3 antibody. Gene expression in the aorta was analyzed by measuring relative *RNA* levels using qRT-PCR for *Pparγ*, *Mcp1, Irak3* and *Adipoq*. Data are means ± SEM. **P<0.01 and ***P<0.001 HFD-fed compared with SD-fed LDL-receptor deficient mice. Abbreviations: HFD, high fat diet; SD, standard diet.

Figure 6 shows accelerated atherosclerosis in aortic arch of high-fat insulin resistant mice, due to increased macrophage content. Aortic *Pparγ, Adipoq* and *Irak3* expressions were decreased, whereas *Mcp1* expression was increased. *Pparα* expression was not significantly decreased in aorta of HFD-fed mice (data not shown). Increased aortic inflammation in HFD-fed mice was further evidenced by a 1.6-fold increase in *Tnfα* (*P*<0.01) and a 7.2-fold increase in *IL6* (*P*<0.001).

## Discussion

Previous studies have shown that treatment with PPAR agonists could prevent vascular inflammation, atherosclerosis and the development of cardiovascular disease [23]–[25]. In the present study, we showed that rosiglitazone, a PPARγ agonist, could reduce obesity-induced systemic inflammation, insulin resistance, macrophage accumulation and atherosclerotic plaque formation. In contrast, treatment with fenofibrate, a PPARα agonist, did not reduce systemic inflammation or plaque formation.

PPARγ agonists not only increase peripheral insulin sensitivity but also cause dramatic decreases in systemic inflammation. In obese diabetic patients, rosiglitazone has been shown to decrease circulating C-reactive protein and IL6 levels [26], [27]. Rosiglitazone, but not fenofibrate, also increased adiponectin in our DKO mice. Previously, we demonstrated a relation between adiponectin and Pparγ in the heart, insulin sensitivity, and cardiac function, independent of cholesterol and triglycerides [28]. PPARγ increases adiponectin through a PPAR-responsive element in the adiponectin promoter in adipocytes [29], [30]. The increase in adiponectin may explain the more pronounced increase in *Pparα* expression in adipose and aortic tissues [31], [32]. Our study also confirmed that adiponectin is essential for the indirect vascular protective effect of rosiglitazone [33]–[35] at least partially through upregulation of Irak3 in plaque macrophages. Indeed, we observed that high levels of adiponectin increases the expression of Irak3 in BMDM, which at its turn is necessary for the indirect vascular protective effect of rosiglitazone on macrophages as evidenced by the elevated Mcp1 protein secretion in IRAK3^−/−^ BMDM *in vitro*. Several recent studies have demonstrated that IRAK3 regulates critical aspects of innate immunity, including the development of endotoxin tolerance after adiponectin exposure. Adiponectin induces IRAK3 expression through ERK1/2 and PI3K/Akt signaling cascades [9]. Furthermore, IRAK3 is an important regulator by which tumor-associated macrophages mimic the phenotype of alternatively activated (M2) macrophages [36]. IRAK3 also attenuates post-infarction remodeling by protecting the heart from uncontrolled inflammation and excessive matrix degradation [37]. A surprising finding was that aortic expression of *Irak3* in HFD-fed insulin resistant mice was observed in association with increased blood levels of adiponectin. But aortic *Adipoq* expression was decreased in association with lower *Pparγ* expression, suggesting that local expression in macrophages is more relevant than systemic expression by adipose tissues. This is in agreement with the observation that rosiglitazone inhibited monocyte/macrophage adhesion through *de novo* adiponectin production in human monocytes [38]. Also, HFD-fed mice had elevated levels of leptin that together with high insulin induces inflammation [39], [40]. Previously, it has been suggested that PPARγ guarantees a balanced and adequate production of secretion from adipose tissue of adipocytokines such as adiponectin and leptin, which are important mediators of insulin action in peripheral tissues [41]. Our data in HFD-fed mice indicate that imbalanced production does not necessarily imply high leptin and low adiponectin. Elevated leptin, even in presence of high adiponectin, is sufficient to generate an inflammatory response.

Similar to PPARγ agonists, PPARα agonists, like fenofibrate, have also demonstrated anti-inflammatory properties in addition to their other beneficial effects on metabolism [15], [42]. However, in our study, fenofibrate treatment had no effect on systemic inflammatory markers, and did not increase adiponectin levels and *Irak3* expression and did not decrease the expression of *Mcp1*. In addition, exposure of IRAK3^−/−^ BMDM to fenofibrate resulted in an increased secretion of Mcp1 protein *in vitro*. The different Mcp1 secretion after rosiglitazone and fenofibrate treatment is independent of activation of the canonical NFκB signaling pathway but is possibly due to the increased ROS production observed after fenofibrate treatment. Indeed, it has been suggested that ROS production is involved in the regulation of *Mcp1* expression [43]. However, we have to take into account that a mouse model is not the best model to evaluate PPARα selective compounds for their anti-atherosclerotic efficacy because in humans fenofibrate also influences lipid metabolism through modulation of cholesteryl ester transfer protein [44]. In diet-fed hamsters, the anti-atherosclerotic efficacy of fenofibrate occurred primarily *via* reductions in pro-atherogenic lipoproteins. However, these hamsters were not obese and did not display insulin resistance [45]. Furthermore, the dosages of fenofibrate and rosiglitazone were approximately 2 and 6 times higher than the maximum recommended daily dose in humans. However, in this study, fenofibrate and rosiglitazone were used as experimental tools to identify underlying mechanisms that could apply to patients treated with the drug. Finally, our data are in agreement with previous data of Duez *et al.* showing that fenofibrate did not reduce atherosclerotic lesion area in the aortic sinus of ApoE deficient mice [46]. However, they found a reduction of cholesterol content in descending aortas of treated mice, an effect that was more pronounced in older mice exhibiting more advanced lesions. Unfortunately, in our study we did not measure cholesterol levels in the descending aorta. Interestingly, fenofibrate reduced lesions in the aortic sinus of ApoE deficient mice overexpressing human apolipoprotein-A1 [46].

### Conclusions

**Figure 7:**
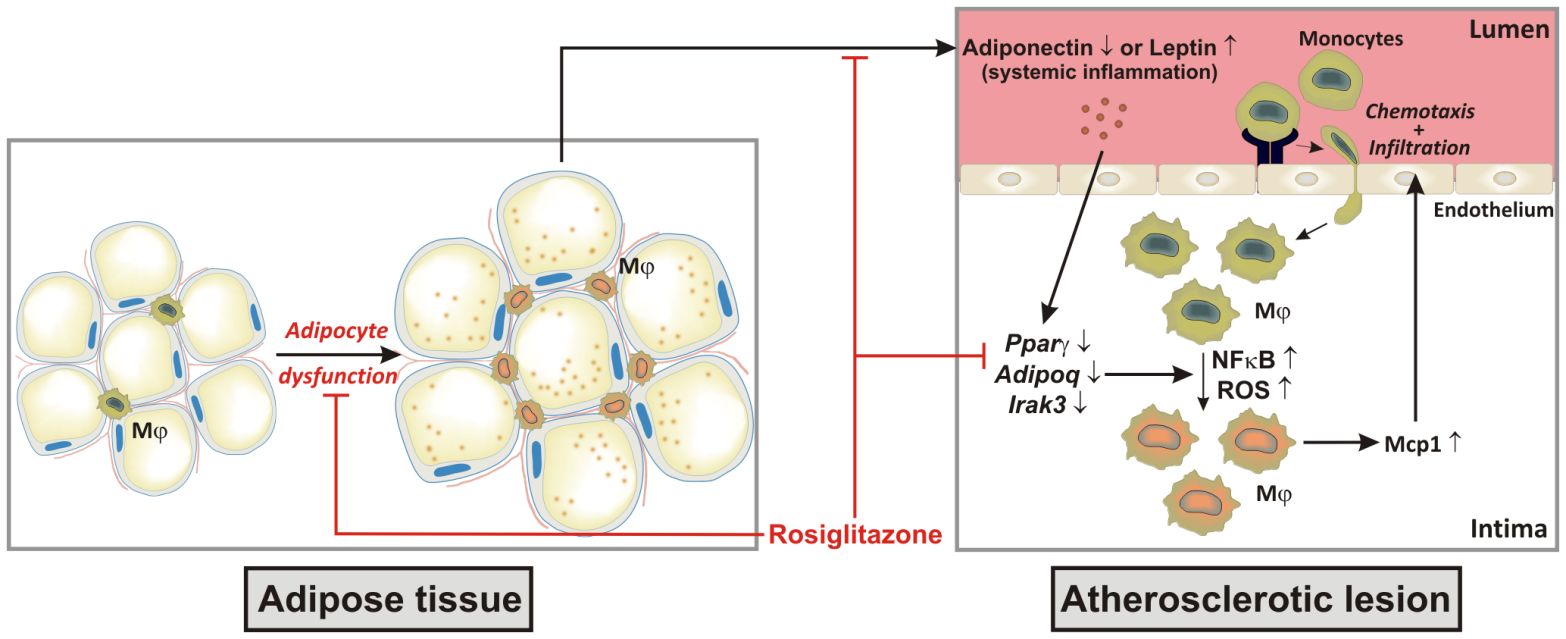
Adiponectin and macrophage-associated Irak3 are indispensable molecules in the anti-atherosclerotic properties of PPAR agonists. The schematic draw demonstrates the anti-atherosclerotic properties of the PPARγ agonist rosiglitazone. Treatment with rosiglitazone improves the adipocyte function characterized by a decrease in adipocyte size, a reduction in adipose tissue macrophages and an increased expression of anti-inflammatory adiponectin. The increase in blood adiponectin and *de novo* adiponectin production in atherosclerotic lesions is necessary for the upregulation of Irak3 in plaque macrophages, which is crucial for the indirect rosiglitazone-mediated decrease in Mcp1 secretion. Abbreviations: Mφ, macrophages; ROS, reactive oxygen species.

In summary, we found that rosiglitazone in contrast to fenofibrate improves insulin sensitivity and adiponectin levels and inhibits inflammation and atherosclerotic plaque formation by reducing M1 macrophage accumulation in the vascular wall. In addition, the adiponectin-dependent upregulation of Irak3 in macrophages is necessary for the reduction in Mcp1 secretion, and thus switch from M1 to M2. HFD-induced increase in leptin causes inflammation associated with decreased *Irak3* expression in association with accelerated atherosclerosis, even in presence of high blood levels of adiponectin but decreased plaque expression of *Adipoq*. In summary, we showed that Irak3 as an inhibitor of NFκB and ROS production is required for the protective action of particularly the PPARγ agonist (Figure 7).
